# Dihydropyridine Reductase Deficiency: Acute Encephalopathy Related to Folinic Acid Treatment Interruption in a Girl

**DOI:** 10.1055/s-0042-1756661

**Published:** 2022-09-19

**Authors:** Maria Grazia Pappalardo, Alessandra Di Nora, Andrea Giugno, Concetta Meli, Annamaria Sapuppo, Piero Pavone, Agata Fiumara

**Affiliations:** 1 Department of Clinical and Experimental Medicine, University of Catania, Catania, Italy; 2 Unit of Clinical Pediatrics, AOU “Policlinico,” PO “G. Rodolico,” University of Catania, Catania, Italy; 3 Child Neurology and Psychiatry Unit, Department of Clinical and Experimental Medicine, University of Catania, Catania, Italy

**Keywords:** encephalopathy, metabolic disease, genetic disorder

## Abstract

We reported the case of acute encephalopathy related to colonic acid treatment interruption in a 12-year-old female child presenting to our unit with episodes of vomiting, headache, irritability, acute confusional state, seizures, and left lower limb hypotonia. Brain magnetic resonance imaging (MRI) showed signs of vasogenic and cytotoxic edema at the cerebellar level bilaterally, and lesions at the temporo-occipito-parietal right level, temporomandibular left, and right thalamic with swelling of the convolutions and reduced differentiation between white and gray matter. The patient had suspended the folinic acid treatment at least 6 months before the present admission. The relation between the clinical signs presented by the girl and folic acid deficiency was confirmed by the result of laboratory assessment and by the answer to the notable clinical improvement with the renewal of folinic acid treatment. Dihydropteridine reductase (DHPR) deficiency is a rare autosomal recessive genetic disorder caused by the quinoid dihydropteridine reductase (QDPR) gene mutations. DHPR deficiency impairs the synthesis of the tetrahydrobiopterin (BH4), an essential cofactor for the hydroxylation of the aromatic amino acids phenylalanine, tyrosine, and tryptophan. When not precociously treated, the disorder may present whit severe neurologic impairment including developmental delay/intellective disability (DD/ID), microcephaly, seizures, movement disorders, cerebral palsy, and other neurological impairments. The clinical and neuroradiologic anomalies observed in our case were unusual, with signs previously unreported in patients with folic acid deficiency. The present case shows that the clinical presentation and MRI anomalies of the cerebral folic acid deficiency may be various and unusual compared with those reported in the literature, and it confirms the usefulness of the continuation of folinic acid treatment during the course of the disorder in patients with DHPR deficiency.

## Introduction

Dihydropteridine reductase (DHPR) deficiency is a rare autosomal recessive genetic disorder caused by the quinoid dihydropteridine reductase ( *QDPR* ) gene mutations located in Ch. 4p 15.32. *QDPR* gene missense mutations, insertions/deletions, and splice site mutations have been reported in patients with this disorder. 1 2 3 4 5 6 The gene encodes the enzyme DHPR, which catalyzes the NADH-mediated reduction of quinoid dihydrobiopterin, an essential component of the pterin-dependent aromatic amino acids hydroxylating systems. DHPR deficiency impairs the synthesis of the tetrahydrobiopterin (BH _4_ ), an essential cofactor for the hydroxylation of the aromatic amino acids phenylalanine, tyrosine, and tryptophan, the deficiency of which causes: (1) increased levels of phenylalanine in the blood (hyperphenylalaninemia [HPA] type 2) and in other tissues and (2) reduced cerebrospinal fluid (CSF) synthesis of neurotransmitters (dopamine and serotonin) and CSF folic acid. 5 6 Clinical manifestations may be observed since infancy, affecting equally both genders and all racial and ethnic groups. Increased level of HPA found through neonatal metabolic screening program may be a clue to this metabolic disorder, and early treatment may prevent severe brain involvement. 7 When not precociously treated, the disorder may present with a series of neurologic disorders including progressive developmental delay, microcephaly, seizures, speech impairment, movement dysfunction, and spastic diplegia or tetraparesis. 8 9 10 11 DHPR deficiency is an extremely rare disorder and accounts for about one-third of all forms of BH _4_ deficiencies, which are estimated to occur in 1 to 2% of all patients with HPA. 9 The incidence of HPA is estimated to be ¼,500 and BH _4_ deficiency identified by the Neonatal Screening Program of the State of Minas Gerais is reported in 2.1 × 1,000,000 live birth with a frequency of 1.71% among HPA patients. 7 The treatment of DHPR deficiency includes the early lowering of blood phenylalanine by dietary restriction, BH _4_ supplementation, and administration of methylfolate or folinic acid and of neurotransmitter precursors such as L-DOPA/carbidopa and 5-hydroxytryptophan. Long-acting dopamine agonists are also utilized in the treatment of this disorder. 11 12 13 14 15

We reported here a 12-year-old girl who was previously hospitalized for a few days to a local pediatric emergency section for two short episodes of seizures. One month after the discharged, she came to Pediatric Department of Catania University (Italy) presenting with episodes of vomiting, headache, irritability, acute confusional state, seizures, and left lower limb hypotonia; she was known to be affected by DHPR deficiency and in treatment since infancy. A thorough personal history revealed that the girl, with the agreement of her parents, had suspended the folinic acid treatment due to poor compliance since at least 6 months before the present admission. The relation between the clinical signs presented by the girl and folic acid deficiency was confirmed by the results of laboratory assessment and by the answer to the notable clinical improvement with the renewal of folinic acid treatment.

This clinical report points out the following: the rarity of DHPR deficiency disorder; the peculiar neurologic manifestations presented by the girl with signs previously unreported in patients with folic acid deficiency; and the confirmation of the usefulness of the folic acid treatment along the course of the disorder in patients with DHPR deficiency as to avoid further neurologic complications.

## Case Report

A 12-year-old female patient was the second born from healthy unrelated parents. Her 7-year-old younger brother is healthy. Family history is negative for neurological disorders. The girl was born at the 39th week of gestation by normal delivery. The mother mentioned to have felt fetal movements normally and did not suffer febrile disorders throughout the pregnancy. Personal history of the girl started at the neonatal period, since at the neonatal screening program for inherited metabolic disorders a positivity for high level of phenylalaninemia (PHA) was found. On admission at Pediatrics Department of Catania University, Neonatal Section, a diagnostic work-up for PHA was started, and a serum phenylalanine level ranging between 492 and 756 nmol/L was reported. A diagnosis of DHPR deficiency was performed, and based on BH _4_ loading test, measurements of urinary, serum, and CSF pterins, DHPR activity in dried blood spots, and analysis of neurotransmitter metabolites in urine and CSF were conducteds. Treatment was started with phenylalanine restriction diet, levodopa/carbidopa (2 mg/kg/day) and 5-hydroxytriptophan (10 mg/mL), and folinic acid (3 mg/day). The girl has been followed up since birth in ambulatory care by the doctor team of the regional center for inherited metabolic disorders and by pediatric neurologists of this department. The girl's growth was normal, and anthropometric measurements were just below the normal range for her age. During the course of the disease, physical examination was normal and no malformation anomalies were found. The thorax, heart, and abdominal organs were normal. At the age of 2 years, the stage of neurodevelopment was delayed as regards motor and language skills. Doses were adjusted according to the age of growth. At the scholastic age, the girl attended school regularly but with poor benefit and with support. At the age of 8 years, she showed an IQ of 56 at the Wechsler Intelligence Scale for Children (WISC)-III test. Laboratory analysis performed along the course of the disease displayed normal electrolytes, plasma and urine amino acids including phenylalaninemia, plasma purine, thyroid and celiac markers, and sialotransferrin.

At the present age of 12 years, the girl came to a pediatric emergency of a local hospital due to the sudden onset of afebrile focal epileptic seizures lasting a few minutes, which was preceded by frontal headache. After 2 hours, the seizures reappeared, lasting again a few minutes with spontaneous resolution. Computed tomography (CT) scan was normal and EEG recording showed basic theta activity at 5 to 6 c/s on the posterior regions and at hyperventilation high-voltage activities and intermittent light stimulation at 20-Hz frequency on the posterior region bilaterally. Levetiracetam therapy at the initial dosage of 500 mg/day (18 mg/kg/day) was started, and she was discharged by the hospital in good condition. About a month later, the girl presented with new sudden episodes of acute blurred consciousness, vomiting, headache, and again epileptic seizures lasting a few minutes and was admitted to the Pediatric Department, University of Catania, Italy. On admission, the general condition of the girl was unsatisfactory: refill time, 2 seconds; weight, 27 kg; height, 132 cm; and head circumference, 49 cm—all within the 3rd percentile. At physical examination, the girl showed confusional state, with poor behavioral interaction and no seizures. She was able to reply to the question with poor and dull voice. Cranial nerves were normal. She was unable to walk without support because of hypotonia involving the left lower limb. Meningeal signs were absent with patellar tendon reflexes present and brisk. Routine laboratory analyses were normal including electrolytes, plasma and urine amino acid, vitamin B _12_ , organic acid, thyroid and celiac markers, sialotransferrin, plasma purine, and total cholesterol. Systemic lupus erythematosus panel, proteins C and S, factor V Leiden, antiphospholipid antibodies, and antithrombin III and coagulation profile as well as inflammatory markers and urinalysis were in the normal range. Plasma PHA was 56 nmol/L (normal range, 25–100 nmol/L), and plasma folic acid was 7.5 nmol/L (normal range, 3.89–26.8 nmol/L). CSF analysis gave the following results: cultures were normal, PHA 60 nmol/L (normal range, 23–95 nmol/L), 5-5-Hydroxyindoleacetic acid (HHA) 34 nmol/L (normal range, 84–360 nmol/L), Homovanillic Acid (HVA) 86 nmol/L (normal range, 140–780 nmol/L), 5-MTHF 97 nmol/L (normal range, 150–170 nmol/L), BH _4_ 24 nmol/L (normal range, 18–40 nmol/L), and N-PHA 22 nmol/L (normal range, 5–25 nmol/L). Otoacoustic examination, electrocardiogram (ECG), and echocardiogram were normal. A new EEG recording showed medium focal time slowdown specifically in the left centrotemporal and right temporo-occipital region and a generalized slow activity. Brain magnetic resonance imaging (MRI) showed the presence of altered signal areas at the cerebellar level bilaterally with diffusion restriction as for vasogenic and cytotoxic edema. Other lesions of the altered signal were seen at the temporo-occipitoparietal right level, with swelling of the convolutions and reduced differentiation between white and gray matter, temporomandibular left, and right thalamic ( Fig. 2A–C ). In consideration of the interrupted folinic acid treatment, as informed by her family members and again confirmed by the laboratory assessment, and excluding the stroke nature of the brain MRI lesions, a new cycle of intravenous folinic acid treatment was started (initially at the dosage of 10 mg/day and then 15 mg/day orally) associated with dexamethasone therapy resulting in progressive clinical benefit. The daily dosage of levetiracetam was also increased. No episodes of headache, vomiting, seizures, or other neurologic signs were observed, and progressive regain of consciousness and improvement in the left lower limb hypotonia were observed albeit with residual hypoesthesia. A new brain MRI performed after 2 months from the admission showed a notable improvement of the lesions previously reported ( Fig. 3A–C ). At 1-year follow-up, the girl had good clinical condition. No other similar episodes were registered. She can walk normally but scholastic performance is poor and IQ test persisted at the precedent levels.

**Fig. 1:**
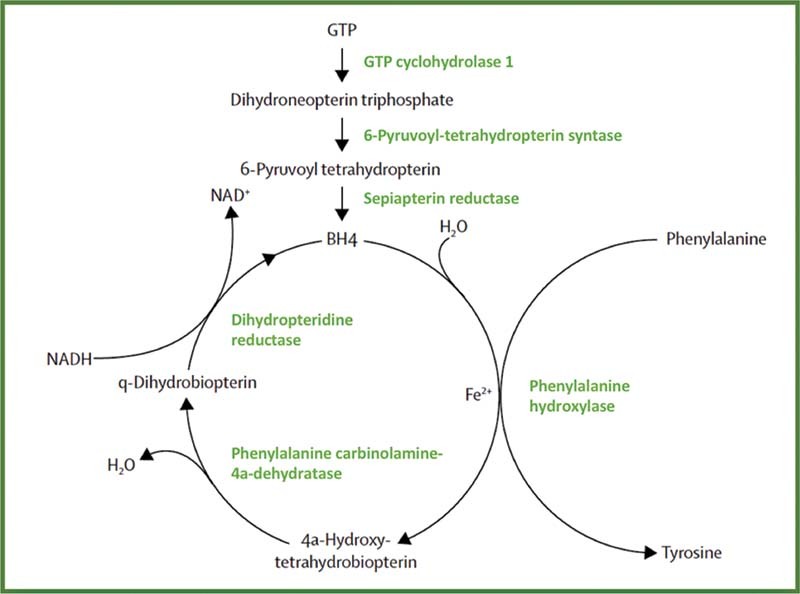
Phenylalanine hydroxylating system. BH _4_ , tetrahydrobiopterin; GTP, guanosine triphosphate. (Reproduced with permission from van Spronsen et al. 13 )

**Fig. 2:**
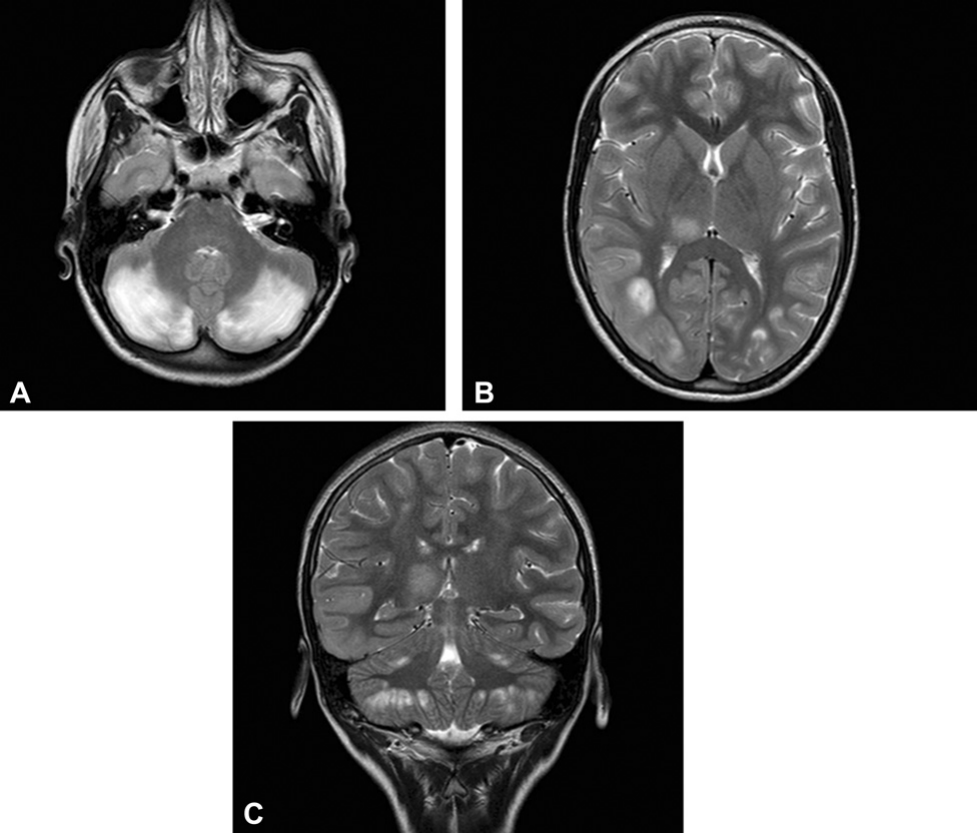
( **A–C** ) MRI images related with acute neurological symptoms.

**Fig. 3:**
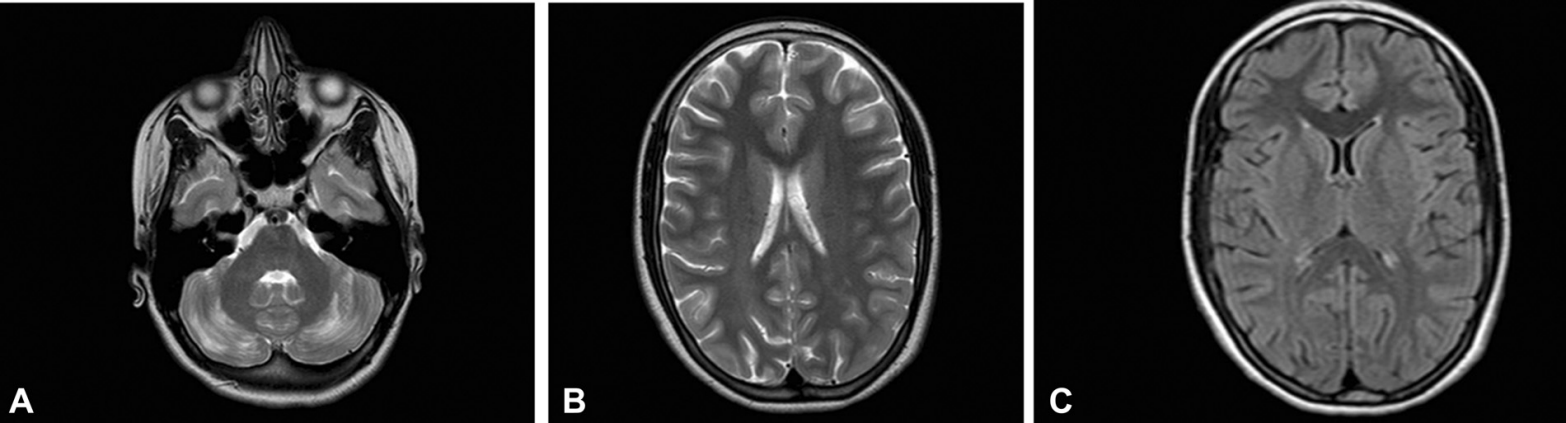
( **A–C** ) MRI imaging performed after reintroduction of folinic acid and adjustment of antiepileptic therapy.

## Discussion

In our patient, DHPR deficiency was diagnosed in the neonatal period through the presence of a high level of PHA at the neonatal screening program and subsequently by appropriate diagnostic laboratory assessment including blood and CSF results. Soon after the diagnosis of DHPR deficiency, the girl was treated and followed up according to the classical protocol for DHPR deficiency. During the course of the years, the treatment was effective, as no complaints were reported with the exception of poor growth and poor scholastic performance. The first clinical episode happened at the age of 12 years and was characterized by a sudden onset of headache and by a focal epileptic seizure lasting a few minutes not preceded by febrile temperature and followed by a second epileptic episode presenting with the same features. Initially, the clinical signs and EEG recording pointed toward the diagnosis of focal epilepsy. One month later, further clinical manifestations occurred with features of acute encephalopathy with headache, vomiting, confusional state, and seizures and admission to the Pediatric Department of Catania University. The brain MRI allowed us to exclude the diagnosis of acute arterial ischemic or thrombohemorrhagic stroke together with the negativity of specific laboratory analysis for this disorder. The information given by the parents on the interruption of the folinic acid treatment in the girl and subsequently the results of the blood and CSF laboratory analyses led us to better clarify the origin of the clinical disturbances presented by the girl.

Early and correct treatment for patients affected by DHPR deficiency is essential, as a delay or an inadequate treatment may result in severe neurologic impairment including developmental delay/intellective disability (DD/ID), microcephaly, seizures, movement disorders, cerebral palsy, and other neurological impairments. Bozaci et al 12 reported on nine patients: three affected by DHPR deficiency and six by BH _4_ deficiency. All the three patients affected by DHPR deficiency were females. One of these (patient 7) was referred at the age of 18 months for walking difficulty and at her last visit showed hypotonia, seizures, and slight ID. Another patient (patient 8) showed DD and slight ID. In this patient, hypersalivation, apathy, dysarthria, and gait disturbances became worse when the blood Phenylalanine (PHA) levels were high, particularly when over 6 mg/dL. The third case (patient 9) was diagnosed at the age of 9 years with ID, seizures, and inability to stand, walk, or even sit without assistance. Miladi et al 16 report on seven members of a large family affected by DHPR deficiency. In this family, a sister and four brothers died between the age of 3 and 7 years. In the sister, a neuropathologic exam showed diffuse demyelination throughout the white matter and spongy vacuolation in the subthalamic nuclei, superior cerebellar peduncles, and tegmentum tracts of the brain stem. The anterointernal part of the putamen was completely necrotic with nearly total nerve cell loss. Abnormal vascular proliferation and calcification of the walls of small, medium, and large arteries and veins and diffusely scattered pericapillary and isolated calcospherites were also reported in the necrotic region.

The clinical and neuroradiologic anomalies observed in the present case were unusual. The girl manifested signs of acute encephalopathy, and brain MRI showed diffuse lesions characterized by vasogenic and cytotoxic edema, swelling of the convolutions, and reduced differentiation between white and gray matter. Anomalies were also reported at cerebellar level bilaterally. In this girl, the failure of folic acid as found in spinal liquor and the improvement of clinical and radiological manifestations after the renewed treatment with folinic acid are suggestive of a highly probable correlation between clinical manifestations presented by the girl and folic acid deficiency. It is well known that brain folate has an important role in myelin formation, and this is supported by the fact that brain folate deficiency is manifested by several neurological manifestations. 17 18 19 In nontreated DHPR deficiency patients, the neuroradiological characteristics usually observed consist of brain atrophy, basal ganglia calcification, white matter changes, ventricular dilatation, areas of hypodensity, and global demyelinating signs. 20 Woody et al 21 reported on a child with DHPR deficiency presenting with seizures and a progressive basal ganglia and other subcortical calcifications. After the use of folinic acid, an excellent seizure control was obtained, suggesting either a direct or indirect anticonvulsant effect of this treatment. Sugita et al 22 reported two siblings with HPA due to DHPR deficiency. The siblings were examined using MRI and CT of the brain. MR images showed white matter changes, whereas CT scan showed characteristic calcifications in the basal ganglia and subcortical region bilaterally. In a 16-year-old boy with DHPR deficiency, the brain MRI displayed high-intensity areas in bilateral frontal and posterior deep white matter on fluid-attenuated inversion recovery (FLAIR). 23 Coronal FLAIR image revealed a high signal in pyramidal tracts. The patient clinically showed moderate DD, parkinsonism, and spastic paralysis.

In our patient, the failure to continue the treatment with folinic acid has caused neurological dysfunction manifesting with acute encephalopathy and correlated with severe symptoms including epileptic seizures. The therapeutic intervention with folinic acid achieved a notable clinical and imaging improvement. The present case shows that the clinical presentation and MRI anomalies of the cerebral folic acid deficiency may be various and unusual compared with those reported in the literature, and it clearly confirms the usefulness of the continuation of folinic acid treatment during the course of the disorder in patients with DHPR deficiency.
